# Supplementary material for: Offline impact of transcranial focused ultrasound on cortical activation in primates
Source: eLife. 2019 Feb 12;8:e40541. doi: 10.7554/eLife.40541 (PMC6372282; doi:10.7554/eLife.40541)
Supplement: Transparent reporting form [file elife-40541-transrepform.docx]

***eLife’s* transparent reporting form**

We encourage authors to provide detailed information *within their submission* to facilitate the interpretation and replication of experiments. Authors can upload supporting documentation to indicate the use of appropriate reporting guidelines for health-related research (see [EQUATOR Network](http://www.equator-network.org/%20)), life science research (see the [BioSharing Information Resource](https://biosharing.org/)), or the [ARRIVE guidelines](http://www.plosbiology.org/article/info:doi/10.1371/journal.pbio.1000412) for reporting work involving animal research. Where applicable, authors should refer to any relevant reporting standards documents in this form.

If you have any questions, please consult our Journal Policies and/or contact us: [editorial@elifesciences.org](mailto:editorial@elifesciences.org).

**Sample-size estimation**

- You should state whether an appropriate sample size was computed when the study was being designed
- You should state the statistical method of sample size computation and any required assumptions
- If no explicit power analysis was used, you should describe how you decided what sample (replicate) size (number) to use

Please outline where this information can be found within the submission (e.g., sections or figure legends), or explain why this information doesn’t apply to your submission:

P32. Materials and Methods: *Statistics*

Sample sizes could not be predetermined statistically in the absence of a prior literature reporting relevant expected effect sizes; instead we adopted sample sizes similar to those reported in previous publications detailing (interventional) macaque fMRI studies.

**Replicates**

- You should report how often each experiment was performed
- You should include a definition of biological versus technical replication
- The data obtained should be provided and sufficient information should be provided to indicate the number of independent biological and/or technical replicates
- If you encountered any outliers, you should describe how these were handled
- Criteria for exclusion/inclusion of data should be clearly stated
- High-throughput sequence data should be uploaded before submission, with a private link for reviewers provided (these are available from both GEO and ArrayExpress)

Please outline where this information can be found within the submission (e.g., sections or figure legends), or explain why this information doesn’t apply to your submission:

Number of experiments (and replications): figure 1, p6. Introduction

Replication experiment: p10-11. Results: *Experiment 3, replication of TUS effects on FPC connectivity*

Data exclusion: p32. Materials and Methods: *Statistics*

**Statistical reporting**

- Statistical analysis methods should be described and justified
- Raw data should be presented in figures whenever informative to do so (typically when N per group is less than 10)
- For each experiment, you should identify the statistical tests used, exact values of N, definitions of center, methods of multiple test correction, and dispersion and precision measures (e.g., mean, median, SD, SEM, confidence intervals; and, for the major substantive results, a measure of effect size (e.g., Pearson's r, Cohen's d)
- Report exact p-values wherever possible alongside the summary statistics and 95% confidence intervals. These should be reported for all key questions and not only when the p-value is less than 0.05.

Please outline where this information can be found within the submission (e.g., sections or figure legends), or explain why this information doesn’t apply to your submission:

Statistical analysis methods: p32-34. Materials and Methods: *Statistics*

Raw data presented: Figure 2: target locations for each individual animal. Figure 4: data points from all animals presented in panels c and i. Fig. 6: data points from all animals presented in panel c. Figure 7: T1w structural MRI images presented for two animals. Figure 8: photographs and histological slices are presented for individual animals.

Statistical tests: non-parametric permutation tests reporting true probability: p7, p9-10, p13; mixed-effects analysis of variance reporting for each test F-value, degrees of freedom of the test and error, p-value, Cohen’s d, and confidence interval: p12-14.

The exact value of N is reported for each experiment: p22. Materials and Methods: *Subject details*

Definition of center: p32. Materials and Methods: *Statistics*

Each test was designed either for a stand-alone inference or to validate prior tests; they included all relevant factors and conditions. Statistical inference on the connectional fingerprints was drawn on cosine similarity metrics that take into account the patterns of coupling across all ROIs together. Accordingly, a correction for multiple tests is not applicable.

(For large datasets, or papers with a very large number of statistical tests, you may upload a single table file with tests, Ns, etc., with reference to sections in the manuscript.)

**Group allocation**

- Indicate how samples were allocated into experimental groups (in the case of clinical studies, please specify allocation to treatment method); if randomization was used, please also state if restricted randomization was applied
- Indicate if masking was used during group allocation, data collection and/or data analysis

Please outline where this information can be found within the submission (e.g., sections or figure legends), or explain why this information doesn’t apply to your submission:

P32. Materials and Methods: *Statistics*

All suitable animals available at the time of experimentation took part in this study. Accordingly, there was no pre-selection nor restriction for group allocation. No data was excluded for analysis. Data collection and analysis were not performed blind to the conditions of the experiments.

**Additional data files (“source data”)**

- We encourage you to upload relevant additional data files, such as numerical data that are represented as a graph in a figure, or as a summary table
- Where provided, these should be in the most useful format, and they can be uploaded as “Source data” files linked to a main figure or table
- Include model definition files including the full list of parameters used
- Include code used for data analysis (e.g., R, MatLab)
- Avoid stating that data files are “available upon request”

Please indicate the figures or tables for which source data files have been provided:

All previously unpublished code will be released as part of MrCat (https://github.com/neuroecology/MrCat) upon acceptance under the Apache2.0 license. In line with the Wellcome Centre for Integrative Neuroimaging policies, we will share post-publication data fully. Previous neuroimaging data we collected are already part of PRIMatE Data Exchange (PRIME-DE) hosted by the International Neuroimaging Data-sharing Initiative.
